# Editorial: Rising stars in hydrothermal vents and cold seeps: 2021

**DOI:** 10.3389/fmicb.2023.1297372

**Published:** 2023-10-06

**Authors:** S. Emil Ruff, Brett J. Baker

**Affiliations:** ^1^The Marine Biological Laboratory, Ecosystems Center, Woods Hole, MA, United States; ^2^The Marine Biological Laboratory, Josephine Bay Paul Center for Comparative Molecular Biology and Evolution, Woods Hole, MA, United States; ^3^Department of Integrative Biology, University of Texas at Austin, Austin, TX, United States; ^4^Department of Marine Science, University of Texas at Austin, Austin, TX, United States

**Keywords:** sediment biogeochemistry, chemosynthesis, microbial symbioses, microbiome, extreme environment

Research in the ecology and biogeochemistry of hydrothermal vents and methane seeps are driven—just like all science—by the hard work of early career scientists. To highlight recent work and invaluable contributions of young scholars we wanted to specifically focus on this demographic group in this Research Topic. Thus, the first authors of all nine articles featured here are graduate students, postdocs, and assistant professors at the beginning of their career and almost all senior authors are early- or mid-career principal investigators. The articles featured here span a broad range of environments around the world, from the deep sea, via shallow marine regions to laboratory cultures, using a broad range of methods from biogeochemical measurements to multi-omics.

This Research Topic includes studies on deep-sea hydrothermal vents and cold seeps. These environments remain fruitful for exploring microbial diversity, as they are difficult to access and require costly sampling vessels. As a result surveys of diversity are still an active area of research. In this topic we had two publications that harnessed rRNA-based studies. In Lazar et al. the authors examined microbial diversity in a geothermal mud volcano underlying the hypersaline Urania Basin, which is off the coast of Crete in the Mediterranean Sea. This study revealed distinct shifts in community structures between brines, muds, and sediments samples. They also found a predominance of spore-forming bacteria likely sourced from deep fluids to the volcanoes. These types of ecosystems are a window into the subsurface. Qi et al. compared the intestinal communities of hydrothermal shrimp (*Rimicaris exoculata*) in the central Indian Ridge, and found distinctions in community structures between juvenile and adults. Juveniles mainly harbored Deferribacterota, while in adults Campilobacterota dominated the intestines.

This Research Topic also comprises studies investigating shallow hydrothermal vent ecosystems. In contrast to deep-sea ecosystems these shallow systems have a direct impact on the food webs and biogeochemistry of coastal and surface environments. The Mediterranean and North Atlantic shallow vents that were studied here are thus located in marine areas that are under more anthropogenic influence, but also easier to access than vents in the deep sea. The coastal hydrothermal vent field of Paleochori Bay, off the island of Milos, Greece, is an example of an ecosystem that has been extensively studied and is used as a model ecosystem for processes that occur at less accessible deep-sea vents. Le Moine Bauer et al. carried out a survey of the microbial communities across eight different hydrothermal habitats and used select metabolic marker genes to investigate major biogeochemical processes. They found that microbial diversity and composition are best explained by the thermal gradients, with archaea dominating the highest temperature regimes. Barosa et al. carried out a similar study yet investigated shallow hydrothermal vent ecosystems in the Aeolian Archipelago, Italy. These vent sites comprised highly diverse communities, potentially driven by light and chemical gradients, and were also shaped by the distinct geochemical regimes.

In contrast to the slightly acidic (Barosa et al.) and strongly acidic vents (Le Moine Bauer et al.) in the Mediterranean, the vents off Iceland studied by Twing et al. were alkaline. The communities of these vents were fundamentally different from most marine hydrothermal ecosystems, they comprised hardly any archaea and resembled those of terrestrial hot springs. One of the lineages typical for terrestrial systems was the phylum Bipolaricaulota (aka Acetothermia or OP1). This phylum was also the focus of the work reported by Coskun et al.. The extreme physicochemical conditions at hydrothermal ecosystems often select for communities of low complexity that are dominated by few lineages of highly specialized microorganisms. Coskun et al. studied such natural “enrichment cultures” in terrestrial hydrothermal springs on the Biga Peninsula, Turkey. The team characterized the metabolic capabilities of two major Bipolaricaulota lineages, an autotrophic lineage thriving in low salinity springs and a heterotrophic lineage abundant at high salinity.

Field studies of diversity, distribution, and biogeochemistry have, and will continue to, advance our understanding of the microbial world. However, linking microbial activity and diversity remains at the forefront of discovery in microbial ecology. The growing toolbox to investigate microbial physiology without culturing includes bioorthogonal non-canonical amino-acid tagging (BONCAT). In Krukenberg et al. the authors couple this approach with fluorescence-activated cell sorting (FACS) to determine the which members of the Guaymas Basin (Gulf of California) hydrothermal sediments are involved in polysaccharide degradation (specifically cellulose, chitin, laminarin, and starch). Another powerful approach to understanding physiological activity and interactions in microbial consortia is to obtain enrichments of mixed communities. This is what was employed by Zhu et al. and Benito Merino et al. to understand methane-oxidizing communities. These studies revealed that lineages commonly associated with these communities including Bathyarchaeota, Lokiarchaeota, and Thermoplasmatales are involved in breakdown of organic compounds (Zhu et al.). Benito Merino et al. obtained thermophilic methane-oxidizing enrichments of ANME-1c archaea and their sulfate-reducing syntrophs (Thermodesulfovibrio spp) from Gulf of California hydrothermal sediments, thus extending the thermal range of anaerobic methane oxidation toward 70°C.

The studies in this Research Topic covered a broad range of hydrothermal environments, deep and shallow, marine and terrestrial, natural and artificial. To characterize and understand the diversity, metabolic capabilities, and ecological niches of vent- and seep-associated microbial communities the researchers used a broad array of culture dependent and independent methods. Despite their different approaches, all studies show that a comprehensive understanding of an ecosystem can only be achieved by combining physicochemical and microbiological analyses. Furthermore, the studies are a reminder that we still know so little about these ecosystems that every survey and investigation of these communities represents a substantial and valuable gain in knowledge.

## Author contributions

SER: Conceptualization, Project administration, Writing—original draft. BB: Conceptualization, Project administration, Writing—original draft.

